# Unlocking Green Oxygen's Potential for Planetary Carbon Management

**DOI:** 10.1002/cssc.202501444

**Published:** 2026-03-04

**Authors:** Martin Held, Jan Backmann

**Affiliations:** ^1^ Bioprocess Laboratory D‐BSSE/ETHZ Basel Switzerland; ^2^ F.Hoffmann‐La Roche Ltd. Basel Switzerland

**Keywords:** CCUS, defossilization, green H_2_/O_2_, oxy‐fuel, waste‐to‐energy

## Abstract

With the rise of the hydrogen economy and the necessity of active planetary carbon management, opportunities emerge for utilizing oxygen generated as a byproduct of electrolytic hydrogen production. While oxygen has established uses, this perspective focuses on novel opportunities arising from the substantial increase in oxygen production driven by the growing green hydrogen sector. Today's large‐scale, fossil‐based chemical industry achieves its high efficiency primarily through integrated networks. Green oxygen could serve as a crucial building block for forming efficient networks of the future chemical industry, contributing to planetary carbon management and the development of a nonfossil‐based chemical industry of the future. Crucially, regulatory barriers to the implementation and scaling of green oxygen utilization need to be addressed.

## Graphical abstract

Water electrolysis generates both H_2_ and O_2_, yet industrial focus remains largely restricted to H_2_‐driven reductions. The parallel use of O_2_ offers considerable rewards but remains underexplored. Unlocking this dual‐gas potential necessitates robust cross‐sector communication to identify and scale viable industrial use cases.

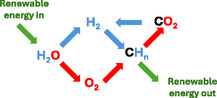

## 1 Introduction

Mitigating climate change necessitates a transition from carbon‐based energy sources to decarbonized alternatives, primarily electrical power generated from renewable sources and hydrogen as a carrier of chemical energy. Importantly, hydrogen's reductive capacity enables the redox reduction of CO_2_ into energy‐rich organic compounds and therefore the synthesis of defossilized feedstocks from CO_2_, a process urgently required for the chemical industry and its downstream users to achieve CO_2_ neutrality [1, 2, 3, 4]. Water electrolysis run on renewable power is the primary method for producing this hydrogen, resulting in the simultaneous generation of the byproduct “green oxygen,” currently largely underutilized and just released into the atmosphere [5, 6].

We have both been concerned with the necessity of supplying defossilized feedstock for chemical production as a means for the reduction of material‐associated scope three emissions, ultimately also enhancing resilience and diversifying supply chains.

Nonfossil feedstock can only be obtained from three different sources: (I) organic recyclates, (II) biomass, and (III) mineral carbon (i.e., CO_2_ or CO). Naturally, we also went into discussions with the hydrogen community, providing H_2_ as chemical energy required, for instance, for redox reduction of CO_2_, and noticed that exploitation of the green oxygen concomitantly synthesized with green hydrogen was barely recognized as a business opportunity. This fact likely stems from the predominant view of hydrogen as a portable carrier for chemical energy for power generation and the transportation sectors that are likely to not be beneficiaries of the byproduct oxygen either now or in future. Due to a clear focus on these fields, intersectoral communication regarding potential applications of “green” oxygen in other sectors remains limited, possibly hindering the identification of—and subsequent investments into—other fields of application.

**FIGURE 1:**
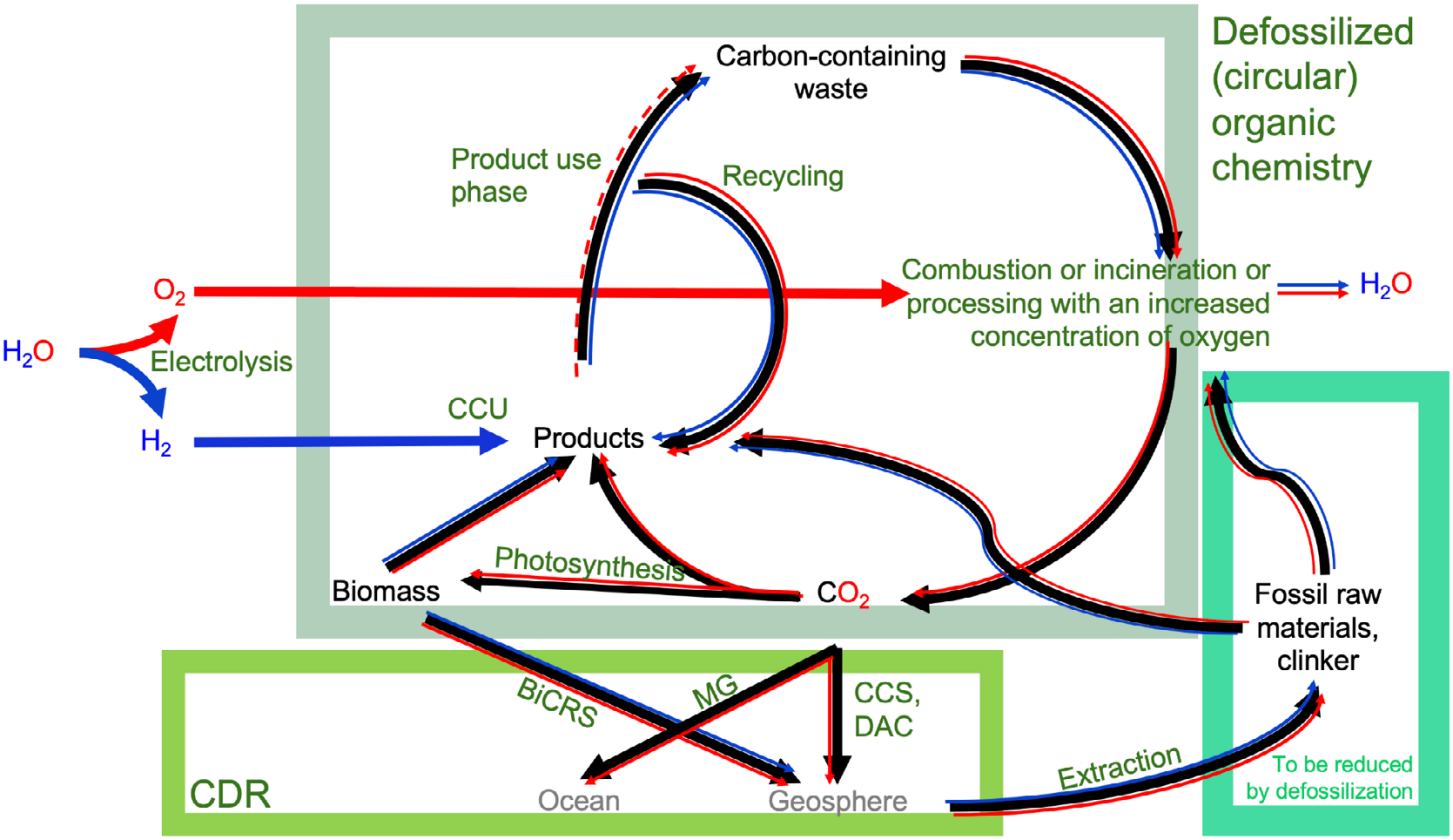
Potential role of green oxygen in future planetary carbon management. Illustration of key pathways for future planetary carbon management (black) referring to the comprehensive and proactive approach of controlling and reducing carbon dioxide (CO_2_) and the role oxygen (red) and hydrogen (blue) from water electrolysis can play. We only show the material‐related carbon flow and are assuming that at the end point of the technological development only renewable, net‐zero energy will be produced. BECCS: Bioenergy with carbon capture and storage, BiCRS: Biomass carbon removal and storage*, CCS: Carbon capture and sequestration, CCU: Carbon capture and utilization, CDR: Carbon dioxide removal, DAC: Direct air capture, MG: Marine geoengineering.

We consulted potential beneficiaries for green oxygen and surveyed the literature to propose applications in environmental protection, circular economy initiatives, and energy efficiency enhancements. According to our findings and as also proposed by a pioneering Japanese study published more than 20 years ago [7] and a more recent study for the UK [8], green oxygen can play an important role in future planetary carbon management (Figure 1) for the control of atmospheric greenhouse gases by technologies enabling decarbonization, defossilization, and carbon dioxide removal (CDR). Consequently, we concluded that the capture and utilization of this oxygen within integrated networks could yield substantial economic and environmental advantages.

Specifically, applications like carbon capture from point sources deemed very costly and energy intensive may become economically more attractive with the availability of green oxygen. Of particular interest for the chemical community is the potential leverage of the supply of defossilized feedstock synthesized from carbon dioxide. As outlined here, this is due to a direct vector resulting from the use of oxygen in the utility‐scale combustion field allowing for reduced energy expenditure for CO_2_ capture from flue gases if used as a starting material for redox reduction and raw material for syntheses. An indirect vector also contributes from the financial returns of the hydrogen sector resulting from the trade of oxygen as a value‐added product.

As a final note, we want to remind the reader that the history of industrial chemistry teaches the lesson that the massive occurrence of any byproduct generally triggers the becoming of entirely new value streams [9]. Therefore, the expanded use of green oxygen is a logical development if care is taken to ensure that the rather reactive gas is safely collected and transported to the point of need.

Open intersectoral dialogs, far‐sighted strategic investments, entrepreneurial courage, technological visionaries, well‐placed governmental programs, and encouraging regulatory policy are needed to develop a positive dynamic and not to miss opportunities arising from the investments currently made in the hydrogen economy.

Although this article often refers to European studies, conditions, and regulations, it is our belief that the principal conclusions are applicable to other regions with similar industrial development.

## 2 Uses of Oxygen to Be Developed or Expanded

Today, oxygen and oxygen‐enriched air mainly find their application in the chemical and metal refining industries and the medicinal sector (see Table S1). However, in the following we provide an overview of possible uses, which, in our view, could become increasingly important due to the massive increase in the availability of green oxygen and upcoming basic technology changes or practices of environmental management.

We envision broader application of oxygen, especially for efficiency improvements in the fields of (I) heat and power generation via combustion and incineration and (II) oxygenation of aqueous systems and briefly visit these two promising application areas.

### 2.1 Generation of Heat and Power by Combustion

Industrialization was largely built upon controlled, large‐scale combustion processes using ambient air as an oxygen source. However, this approach resulted in substantial CO_2_ emissions, leading to significant societal and regulatory pressure on operators worldwide to mitigate them.

A rapidly developing technology to meet this challenge is CO_2_ capturing followed by either storage (carbon capture and sequestration (CCS)) or utilization (carbon capture and utilization (CCU)) [10] However, flue gases from traditional air‐fired processes are inevitably heavily diluted with nitrogen, resulting in low CO_2_ concentrations in the off‐gas. This necessitates energy‐intensive CO_2_ concentration steps, such as amine scrubbing, before transport to storage or utilization sites. There are also significant advancements made in the field of CO_2_‐separation, suggesting that new technologies may be deployed in the next years [11]. However, setting up of CO_2_‐concentration facilities in any case incurs substantial capital and operational costs, thereby significantly impacting potential financial returns during CO_2_ capturing.

Oxy‐fuel combustion (OFC) employing oxygen or oxygen‐enriched gases such as air or recirculated flue gases is a recently conceived alternative to the widely adopted air‐fired processes. While still in the development stage, OFC presents notable benefits, as it potentially allows for reduced boiler heat losses, less spacious designs at comparable performance, and—most importantly—the generation of highly concentrated CO_2_ flue gases.

After the removal of combustion residues (e.g., water, ash, particulate matter, slag, and soot), the flue gas from OFC is indeed of sufficient quality and concentration to directly allow for transport [12, 13, 14]. Yet, currently one of the obstacles for implementing OFC on a larger scale is the availability of oxygen. As a matter of fact, most theoretical OFC studies therefore consider the production of oxygen as an additional step realized by dedicated air‐liquefaction units built near incinerators. In the absence of governmental steering measures, application of oxygen for OFC is therefore, of course, associated with high investment costs (CAPEX) due to additional construction work and high operating costs (OPEX) of the operational units required for contraction of CO_2_, hence elevating the entry barrier.

**FIGURE 2:**
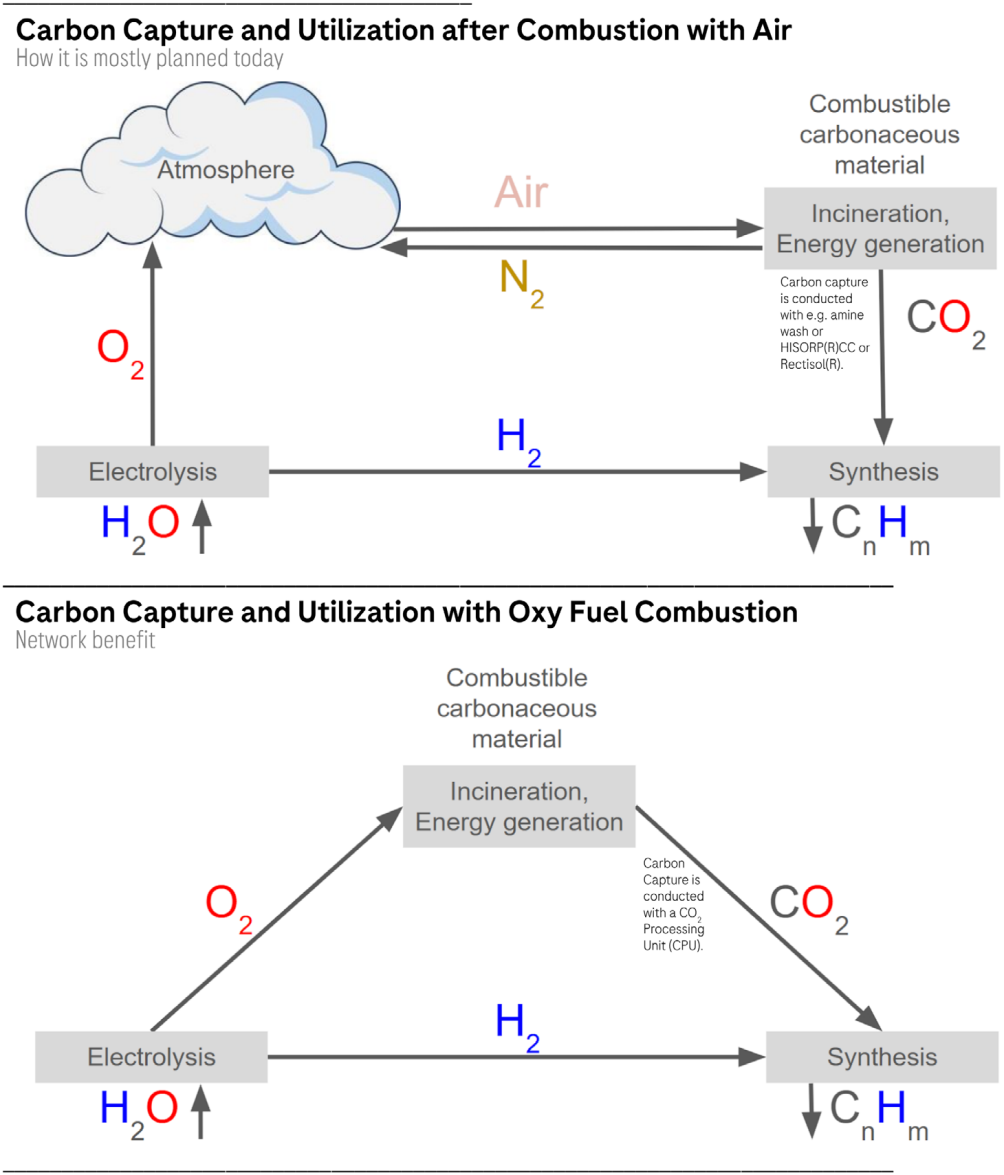
The role of oxygen generated as a byproduct of green hydrogen in CCU. Upper part: schematic depiction of CCU without oxy‐fuel combustion (OFC). Bottom part: Schematic depiction of CCU with OFC. C_n_H_m_ represents any synthesized carbonaceous chemical.

However, we argue that oxygen from electrolysis could be directly used in OFC (see Figure 2). For achieving this goal, the infrastructure should be planned correspondingly to allow efficient use of reductive (H_2_), oxidative (O_2_), and carbonaceous (above all CO_2_) gas streams. Obviously, the highly concentrated carbon dioxide flue gases produced by OFC can also be sequestered directly or concentrated further in a less energy‐intensive way as compared to diluted streams obtained from air‐fired processes.

#### 2.1.1 Waste‐to‐Energy

In the order of 260 Mt of municipal solid waste have been incinerated for energy recovery in 2021 [15]. Due to the huge climate impact of the released flue gases as compared to, for instance, removal of the waste‐bound carbon from the cycle by landfill, waste‐to‐energy processes are under scrutiny, urging the adoption of methods for CO_2_ capturing. As OFC potentially allows for direct carbon capture with no dedicated CO_2_‐concentration step downstream of the combustion process, its adoption can greatly facilitate the transition. This is also reflected by a steadily increasing number of scientific articles dedicated to OFC in the waste‐to‐energy field [16]. Notably, though of smaller climate impact, OFC also presents a potential pathway for high‐temperature thermal treatment of recalcitrant chemical substances such as per‐ and polyfluoroalkyl substances (PFAS) in, for example, waste, neglected deposits, or contaminated soils [17]. However, the thermodynamic constraints imposed by the Boudouard equilibrium at elevated temperatures may lead to undesirable side reactions such as the formation of soot and tar [16]. Therefore, the implementation of OFC in hazardous waste management necessitates careful consideration of operational parameters to optimize destruction efficiency while minimizing the formation of unintended byproducts.

OFC combustion of waste is of value if biogenic fuels (including waste) are incinerated, as the combusted matter has been formed from CO_2_ that has previously been removed by plants from the atmosphere. Postcombustion capturing and sequestration of biogenic CO_2_ therefore allows the generation of carbon‐negative emissions, while the generated heat can still be used directly or for power production [15, 18]. Similarly, using biogenic CO_2_ with green hydrogen and renewable energy allows manufacturing of defossilized feedstocks and—depending on product lifetimes—offers temporary carbon fixation and therefore potentially though transient negative emissions, but at least the supply of net‐zero organic chemicals of plastics. OFC in waste incineration hence offers efficient ways to control and reduce emissions and energy expenses with decisive advantages as compared to air‐fired systems.

#### 2.1.2 Cement Production

OFC can facilitate carbon capture in cement kilns. Due to the high emissions of 1.6 billion t CO_2_/a in 2023 [19], the cement industry is facing intense pressure to implement carbon capture. IEA projections hence consider massive employment of CCUS key for their CO_2_ reduction in this sector [20]. Interestingly, combustion of fuels required for calcination is only responsible for one‐third of the emissions. The rest is due to the mineral CO_2_ released during lime burning. Even if alternative sources for process heat are available (e.g., hydrogen), CO_2_ emission from limestone will therefore remain large. However, while relying on OFC, CO_2_ concentration in the off‐gases can increase due to absence or reduction of nitrogen in the gas fed. To this end, energy savings, especially during subsequent CO_2_ capturing, can be anticipated. This potential, for instance, has been recognized by the four European cement manufacturers Buzzi SpA, Dyckerhoff GmbH, Heidelberg Materials AG, SCHWENK Zement GmbH & Co. KG, and Vicat S.A., which joined forces in 2019 to form the research company CI4C GmbH & Co. KG, aiming at implementation of OFC for facilitated CO_2_ capturing in the project “catch4climate” on the premises of the SCHWENK cement plant in Mergelstetten [21]. Other larger industrial consortia [22] and academic groups [23, 24] are also actively pursuing R&D efforts to benefit from the power of electrolysis for cement manufacturing.

#### 2.1.3 Power Generation

While we are truly hoping that in future large‐scale electricity will come from renewables instead of fossil fuels, the sector is currently emitting in excess of 35 billion metric tons of CO_2_ per year in 2023 [25]. Even if one assumes that a significant part of today's fossil capacities will be replaced by nonfossil sources in the medium term, we realistically assume that billions of tons of CO_2_ will still be emitted in the next decades. Also here, OFC will allow for an increase of the CO_2_ concentration in the flue gas; following an analogous rationale as above, OFC implementation will therefore allow energy savings during CO_2_ capturing in this sector as well.

### 2.2 Oxygenation of Aqueous Phases

The biggest economic and sustainability challenge in the oxygen supply of aqueous systems is energy consumption [26]. This is due to the low water solubility of oxygen (about 8 mg/l), requiring considerable energy input to achieve efficient oxygen transfer from air. However, increasing the oxygen concentration in the feed gas and therefore the oxygen partial pressure at the water–gas interface allows for increased oxygen transfer rates without an energy‐intense increase in compression pressure. Coupling large‐scale units for the supply of oxygen fed to aqueous systems therefore potentially allows for considerable energy savings.

We think that especially the potential benefits of oxygen‐feeding systems to (I) wastewater treatment plants and to (II) open waters deserve attention.

#### 2.2.1 Wastewater Treatment

Globally, in the order of 1000 km [3] of wastewater is generated annually. The electricity required for treatment of processed wastewater is enormous and amounts to 3% of the global electricity production [27]. Data of the municipal wastewater sector indicates further that aeration, as required for stimulation of biological degradation processes, accounts for more than half of the power consumption of wastewater treatment plants. However, the use of oxygen‐enriched gas feeds [28] but also coupling to H_2_/O_2_ networks in general [29] has considerable potential for a reduction of the power expenses.

Unlike other sectors, there is no mandatory requirement for backend CDR efforts, as the emitted CO_2_ is mostly biogenic and therefore largely greenhouse gas neutral. However, CDR efforts can certainly be implemented when negative emissions are desired. Even without CDR, there remains significant potential for energy savings during gas feeding. The gas‐feeding infrastructure is already in place, and blending oxygen into the feed can potentially allow to quickly reduce the amount of pressurized gas and therefore energy expenses.

#### 2.2.2 Open Water Applications

To address oxygen depletion in water bodies, a condition driven mainly by eutrophication and climate change, aeration is a vital remediation approach [30, 31].

In Switzerland, for example, aeration of lakes has led to great success in renaturalizing ecologically overturned surface waters. Similarly, successes could also be achieved with inland seas such as the Baltic Sea, which is suffering from low dissolved oxygen [32, 33]. Large‐scale capacities for electricity generated by offshore wind farms are already available in the Baltic Sea. If electrolyzer capacities are built close by, high volumes of oxygen will become available where they are needed. Due to the short transport distances, efforts toward oxygenation of the Baltic Sea—expected to have a positive impact on both fish yields and environmental health—can be greatly facilitated [34].

In this way, wind farms could view seabed aeration as an ecological contribution to compensating for frequently criticized negative aspects resulting from their activities and thus help to increase acceptance.

Furthermore, use of electrolyzers with oxygen separation and seawater aeration can make the development of the respective systems more attractive and thus also promote commercial use of oxygen. Finally, oxygen can of course also be used on a smaller scale, namely for the aeration of aquacultures, for instance, to maintain optimal dissolved oxygen levels in fish farming, promoting healthier and faster‐growing fish populations.

### 2.3 Technical Realization and Future Projections

In this section, we are sketching potential routes for technical implementation of the use of green oxygen.

#### 2.3.1 Efficient Oxygen Transport and Storage—Leveraging Oxygen in Integrated Industrial Systems

Presently, oxygen is primarily conveyed via high‐pressure gas cylinders and liquid cargo in road vehicles, with pipeline networks representing a less utilized mode of transport. Storage options include compressed gas cylinders or cryogenic tanks for liquid oxygen. With the increasing commercial use of oxygen, especially for carbon management (see Figure 2), the demand for robust logistics and distribution systems would increase significantly. Depending on transport distance, ramping up of expensive high‐pressure infrastructure for pipeline transport may not always be necessary. In any case, infrastructure costs must be carefully considered while weighing the relatively low value of transported oxygen against the relatively high cost of pipeline materials required for safe handling [35, 36, 37, 38].

We envision future oxygen distribution becoming integrated into a comprehensive gas distribution network centered around three key elements:

1. **Hydrogen delivery**: Supplying hydrogen for both decarbonization (replacing fossil fuels as an energy carrier) and defossilization (using hydrogen and CO_2_ as feedstock for low‐carbon emission or net‐zero materials production).
2. **Carbon dioxide transport**: Moving CO_2_ from combustion and incineration sites for either sequestration (CCS) or material utilization via hydrogen‐based redox reduction (CCU).
3. **Oxygen distribution**: Delivering oxygen to sites where oxidation reactions, including combustion and incineration, are performed.

Logistics for the first two streams—hydrogen and carbon dioxide—are already emerging as large‐scale, often international, infrastructure networks. However, they differ in structure and scope. The hydrogen network must be more finely branched, as it needs to serve not only industrial but also small‐scale and even private users. Among the large‐scale industrial consumers, hydrogen is either used for energy generation or the production of materials (e.g., in the synthesis of methane or methanol), the latter being closely tied to the chemical industry, where such products serve as feedstock.

In contrast, the CO_2_ transport network serves two main purposes: CO_2_ sequestration and utilization mainly targeting large emitters. These emitters are facilities operating under controlled conditions and generating high CO_2_ volumes and must be connected to the CO_2_ network to not release the CO_2_ into the atmosphere. In cases where CO_2_ is to be utilized, the receiving site has, of course, also to be connected to the hydrogen network.

The oxygen distribution infrastructure, in contrast, would connect water electrolysis facilities to oxidation sites. These include high‐intensity operations—such as waste incineration—generating concentrated CO_2_ streams suitable for facilitated capture. But smaller‐scale uses, for example, oxygenation in fish farming and other low‐intensity oxidation processes, for example, open‐water aeration and sewage treatment, can also be accounted for. Oxygen transportation hence needs to predominantly serve industrial users but may also satisfy smaller commercial and ecological needs. Given the challenges of transporting and storing oxygen, local and regional distribution systems seemed to be a promising and practical solution to us.

More immediately relevant to the chemical industry is the link between large CO_2_ emitters and CCU sites synthesizing redox‐reduced feedstock (e.g., methanol or methane) as starting material for chemical syntheses. Notably, the chemical sector benefits indirectly from the use of green oxygen due to energy and capital savings in CO_2_ capture processes culminating in cost reductions at the start of the value‐added chain to redox‐reduced carbon synthesized by CCU. Finally, dedicated pipelines for transporting energy‐rich, high‐volume CCU products could further enhance synergies.

We are envisioning that for industrial applications, green oxygen is used most efficiently within integrated networks due to the potentially minimized transport distances [36, 37]. This suggests that for the chemical industry the largest possible benefit can be derived from the combination of the production of green hydrogen accompanied by green oxygen, CCU or CCS combined with process‐heat utilization. Notably, this integrated model mirrors the structure of contemporary fossil‐based industrial networks.

The future viability of a defossilized, material‐converting industry hinges on the effective exploitation of such integrated network advantages. We believe that there are opportunities for retrofitting existing plants that rely on fossil fuels if postcombustion carbon capture is viewed as a bridge measure for climate protection until the final phase‐out of the use of the avoidable fossil fuels, for example, for energy production by combustion. Furthermore, prospects arise for establishing novel integrated sites proximal to sources of surplus renewable electricity and hydrogen electrolysis capabilities. Additionally, new integrated sites may be strategically located near sources of unavoidable CO_2_ emissions, such as cement factories and waste incineration plants.

#### 2.3.2 Projected Future Green Oxygen Production Volumes From Electrolyzers

According to the IEA's Global Hydrogen Review 2025, the global H_2_ volume procured by electrolysis may increase to 28 Mt per year by 2030 [39]. Hence, green oxygen of about 220 Mt O_2_ will become available. An exemplary overlay of the O_2_ production volumes with, for instance, the volumes of terminally treated waste in the European Union [40, 41] as of today (59 Mt of municipal waste plus 45–75 Mt (dry weight) of sewage sludge [42]) and while assuming commercial exploitation of O_2_ for controlled oxidation processes suggests that green oxygen may become a limiting resource in the next years to come. Consequently, the optimal integration of oxygen into operational routines must be thoroughly examined. As, to the best of our knowledge, no study for benchmarking of the potentially rewarding applications of green oxygen has been made yet, we argue that stakeholders from all sectors should unite and develop and advocate action plans.

#### 2.3.3 Availability of Electrolyzers Allowing for Oxygen Production

Our internet research showed that several industrial‐scale water electrolysis systems can be extended to include the option of capturing the coproduced oxygen (see Table S2). How easy this is, how much extension costs, what the operating costs are and how well the oxygen capture presently works were not the subject of our investigations.

#### 2.3.4 CO_2_ Electrolysis and Other Technologies for Future Production of Green Oxygen

Although we did not discuss these technologies here, high‐temperature CO_2_ electrolysis, particularly using solid oxide electrolysis cells (SOECs), offers significant potential for producing green oxygen as a byproduct. Meanwhile, there are even efforts undertaken to conduct CO_2_ electrolysis at low temperatures.

There are also promising technologies that coelectrolyze CO_2_ and H_2_O, also generating oxygen as a byproduct [43]. Furthermore, photoelectrochemical (PEC) reactors, which upon concentrated solar energy input are splitting water to give hydrogen and oxygen as a byproduct, are conceived [39, 44].

It's hence clear that other options for synthesis of green oxygen are likely to mature soon. This is precisely where new network opportunities arise, as discussed above.

## 3 Discussion and Outlook

### 3.1 Business and Economic Aspects

Currently, oxygen is produced as a byproduct of air liquefaction, primarily driven by the profitable extraction of noble gases. However, while growth of air liquefaction follows the general pace of global economic development, the rapidly increasing demand for hydrogen will soon generate large volumes of green oxygen. Recent studies indicate that O_2_ from water electrolysis can be delivered at costs as low as $13/ton if transported by short‐distance pipelines not exceeding 20 km in length, which is thereby almost a quarter of the price of O_2_ from air liquefaction [38].

Furthermore, hydrogen production and, to a similar extent, the closely coupled renewable energy sector are both highly dynamic, and additional quantum leaps and efficiency improvements, and therefore even lower investment barriers, can be anticipated. Therefore, economic actors designing new plants or scaling sustainability technologies that can utilize green oxygen should confidently anticipate its availability and clearly communicate their needs to future suppliers, that is, the hydrogen industry. By coordinating in this manner, producers and users can efficiently align and concurrently scale their designs, including transportation systems like pipelines.

Taken together, these considerations suggest that oxygen from electrolysis has the potential to become a regularly used raw material in various sectors. It is our expectation that the utilization of oxygen in both OFC and water oxidation will have a mutually reinforcing effect, leading to an expanded market for water electrolysis facilities that capture oxygen. To capitalize on green oxygen, different industries need to communicate well, trust each other, and believe in the shift to net‐zero. Imagine a millipede—it needs all its legs working together smoothly for it to move forward efficiently.

### 3.2 Regulatory and Policy Challenges Related to Green Oxygen

The EU promotes and regulates industrial‐scale hydrogen production, which is indirectly also influencing green oxygen availability. However, regulatory requirements, particularly those regarding “additionality,” are dampening production growth, creating administrative burdens, deterring potential users, and delaying projects, thereby impacting both demand and accessibility. It has already been speculated that overly strict criteria could reduce EU hydrogen production by 20%–40% by 2030 compared to flexible scenarios [45].

The “additionality” principle (see the EU's Renewable Energy Directive) mandates that renewable electricity for hydrogen electrolysis must come from new, dedicated facilities. This aims to ensure genuinely green hydrogen and prevent diverting renewable electricity from other beneficial sectors. In practice, this means that electrolysis and renewable power capacities also must be scaled in parallel. To this end, dark doldrum periods leading to low or nil power output from bespoke plants cannot be bridged, thereby putting a risk on the operational stability and—most importantly—not allowing for high‐capacity utilization of electrolyzers. Although these factors are specific to the hydrogen sector—which considers them as barriers to implementing and scaling green hydrogen production—they also hinder the deployment of green oxygen.

Global political and regulatory uncertainties also threaten progress. Implementing sustainability technologies is costly and requires long‐term market stability for an acceptable return on investment. Dynamically changing policies in major economies can therefore hinder efforts to achieve sustainability. Decades of planning security with consistent and improved frameworks are essential, and steadfastness is crucial [46].

To support green oxygen use, linked to green hydrogen production, the regulatory body may consider the following adoptions.

• **Simplify regulatory requirements**: Reduction of the burdens related to “additionality,” temporal and geographic correlation for renewable electricity in hydrogen electrolysis.
• **Allow flexibility in renewable electricity sourcing**: Permission of the use of already available renewable energy for hydrogen electrolysis.
• **Ensure political and regulatory steadfastness**: Maintenance of consistent policy frameworks to encourage investment.

By addressing those regulatory challenges and providing a supportive policy environment, the EU Green Industrial Deal can significantly promote green oxygen production.

### 3.3 Concluding Remarks on Oxygen and Carbon Cycles in Biosphere and Technosphere

Oxygen, carbon, and hydrogen derived from water and carbon dioxide are critical for controlled generation, storage, and utilization of chemical energy in both technological and biological systems. In the biosphere, the three elements C, O, and H required for the assembly of reduced and oxidized carbon circulate between autotrophs and heterotrophs, frequently at low CO_2_ and moderate O_2_ concentrations and generally at modest reaction and volumetric energy turnover rates.

**FIGURE 3:**
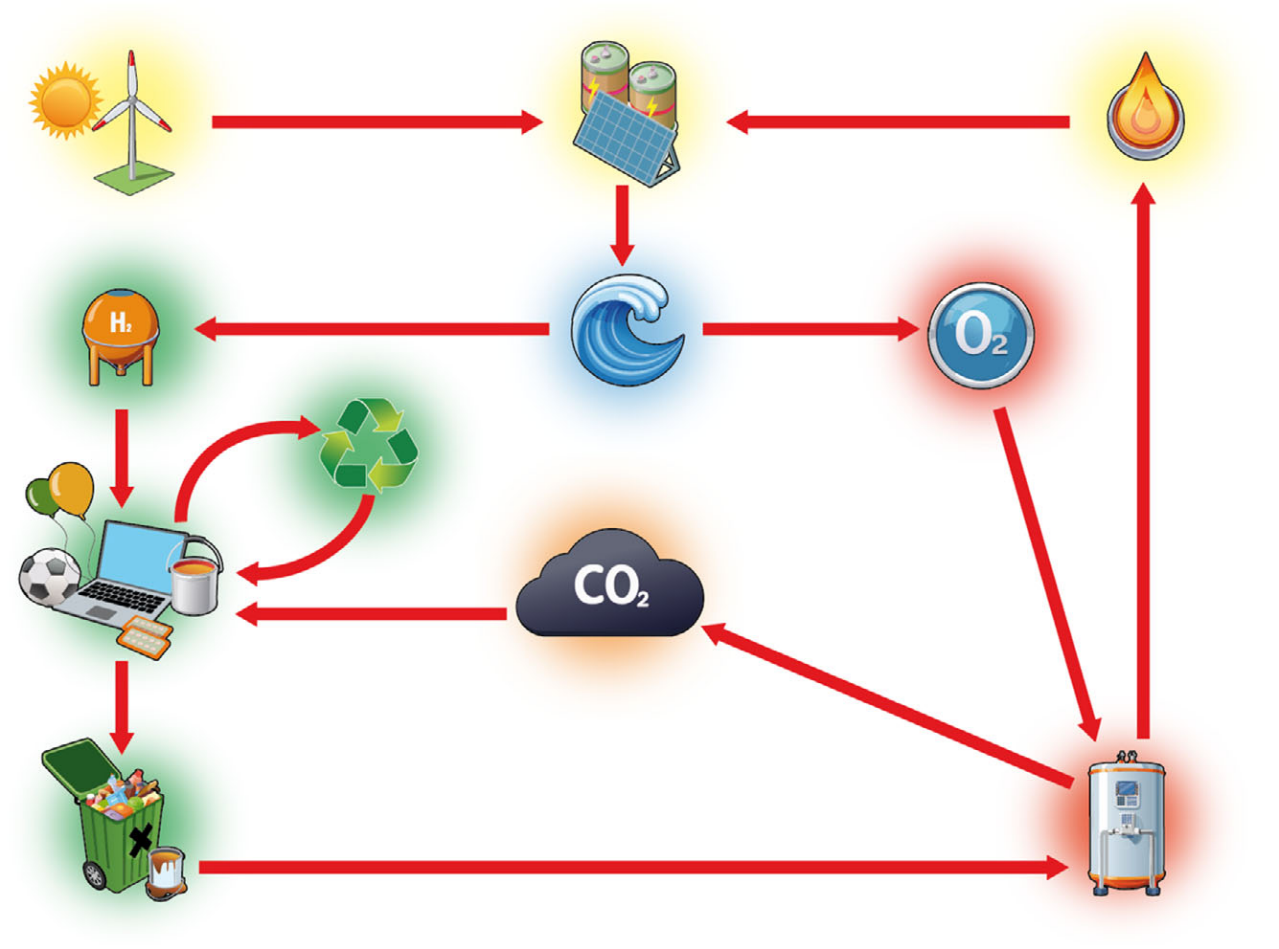
Cycling of carbon required by the chemical industry as feedstock: Electricity from renewables and waste‐to‐power processes is used for electrolysis of water to hydrogen and oxygen. Hydrogen serves as a reductant for converting CO_2_ into feedstock for the chemical and other industries. Use of the comanufactured oxygen in waste‐to‐power plants improves combustion control, reduces oxidant supply energy, and increases CO_2_ concentration in flue gas, thereby significantly lowering the energy demand for CO_2_ capturing and its redox reduction to feedstock for industrial use.

In contrast, industrial processes are generally carried out with concentrated substances and at high reaction rates, with one presupposing the other. The use of chemically pure or well‐defined mixtures coupled with very high reaction intensities compared to the biosphere is therefore both an enabling and a defining feature of the technosphere. Global efforts to synthesize hydrogen as a chemical reducing agent with simultaneous production of the complementary oxidant oxygen should be considered in this context and recognized as an opportunity to further improve the efficiency of technospheric processes, especially in areas where chemical energy (see Figure 3) is released or required for product synthesis.

If orchestrated in the right way, the availability of concentrated green oxygen hence provides an important puzzle piece for industry, hydrogen producers, and large‐scale CO_2_ emitters, as it allows them to collaboratively move toward new operational optima. Essentially, using green oxygen might allow us to better separate and isolate the anthropogenic technosphere from the broader planetary ecosystem.

## Supporting Information

Additional supporting information can be found online in the Supporting Information section. **Supporting** **Table** **S1**: Selected established uses of oxygen and air as oxidant for industrial and medicinal purposes under controlled conditions. **Supporting Table S2**: A selection of manufacturers (in alphabetic order) that produce water electrolysers, which also allow the capturing of oxygen.

## Conflicts of Interest

The authors declare no conflicts of interest.

## Supporting information

Supplementary Material
