# Supplementary material for: Unlocking Green Oxygen's Potential for Planetary Carbon Management
Source: ChemSusChem. 2026 Mar 4;19(5):e202501444. doi: 10.1002/cssc.202501444 (PMC12961174; doi:10.1002/cssc.202501444)
Supplement: Supplementary file 1 — Supplementary Material [file CSSC-19-e202501444-s001.pdf]

Supplementary Material

Table 1. Selected established uses of oxygen and air as oxidant for industrial and medicinal purposes under controlled conditions

Use	Description
Chemical processes	Oxygen is an essential component in various chemical reactions, oxidation, and synthesis processes - including glass and ceramic manufacture, pulp and paper production, petroleum processing and pharmaceutical manufacturing.
Medical Applications	Oxygen is commonly used in hospitals and clinics to support patients with respiratory conditions. It can be used for oxygen therapy, anesthesia, and life support systems.<br>The COVID-19 pandemic significantly highlighted the importance of medical oxygen infrastructure, leading to new technologies and applications.
Steelmaking and metals refining	Basic Oxygen Steelmaking (BOS): In this process, pure oxygen is blown into a bath of molten blast-furnace iron and scrap. The oxygen reacts with impurities such as carbon, silicon, phosphorus, and manganese, oxidizing them and helping to refine the iron into steel.<br><br>Oxy-Fuel Welding and Cutting: This method uses a combination of oxygen and a fuel gas (such as acetylene) to produce a high-temperature flame. The flame is hot enough to melt metals, allowing them to be welded together or cut apart.<br><br>Oxygen-Enriched Air in Smelting: In various smelting processes, oxygen-enriched air is used to increase the efficiency of the reactions. This is common in the production of non-ferrous metals like copper and nickel.
Biotechnology and Fermentation	Mammalian Cell Culture: Oxygen is essential for the growth and maintenance of aerobically grown cells in culture. It is typically supplied through aeration in bioreactors or incubators.

Table 2. A selection of manufacturers (in alphabetic order) that produce water electrolysers, which also allow the capturing of oxygen.

This information is compiled to the best of our knowledge. We assume no responsibility that the systems offered meet any specific technical and regulatory requirements. This list does not claim to be complete.
This list primarily features established manufacturers. It's worth noting that numerous promising startups are actively developing innovative electrolyzer technologies, including membrane-less designs and advanced materials. Therefore, the field is dynamic, and it's highly likely that new suppliers will enter the market in the near future.

**Company**	**Link**
Andritz	https://www.andritz.com/products-en/group/metals/green-hydrogen/ael
Bloom Energy	https://www.bloomenergy.com/bloomelectrolyzer
Cummins Inc	https://www.cummins.com/sites/default/files/2023-10/electrolyzer-brochure.pdf
Hygreen Energy	https://www.hygreenenergy.com
ITM Power	https://itm-power.com/
John Cockerill	https://hydrogen.johncockerill.com/en/products
Linde	https://www.linde.com/clean-energy/our-h2-technology/electrolysis-for-green-hydrogen-production
LONGi	https://www.longi.com/en/products/hydrogen
McPhy Energy	https://mcphy.com/en
Nel Hydrogen	https://nelhydrogen.com/water-electrolysers-hydrogen-generators/
Plug Power, Inc.	https://www.plugpower.com
Quest One	https://www.questone.com/en/products/detail/mhp-series/mhp-electrolyser/
Siemens Energy	https://www.siemens-energy.com/global/en/home/products-services/product-offerings/hydrogen-solutions.html
Toshiba Energy Systems & Solutions Corporation	https://www.global.toshiba/ww/news/energy/2023/10/news-20231030-01.html

Thyssenkrupp Nucera	https://www.thyssenkrupp-nucera.com

See also:
https://www.blackridgeresearch.com/blog/list-of-global-top-hydrogen-electrolyzer-manufacturers-companies-makers-suppliers-in-the-world

https://www.techsciresearch.com/blog/top-electrolyzer-manufacturers-leading-the-green-hydrogen-revolution/4570.html
